# Role of Mitochondria in HIV Infection and Associated Metabolic Disorders: Focus on Nonalcoholic Fatty Liver Disease and Lipodystrophy Syndrome

**DOI:** 10.1155/2013/493413

**Published:** 2013-07-21

**Authors:** P. Pérez-Matute, L. Pérez-Martínez, J. R. Blanco, J. A. Oteo

**Affiliations:** ^1^HIV and Associated Metabolic Alterations Unit, Infectious Diseases Department, Center for Biomedical Research of La Rioja (CIBIR), Piqueras 98, La Rioja, 26006 Logroño, Spain; ^2^Infectious Diseases Department, Hospital San Pedro, La Rioja, 26006 Logroño, Spain

## Abstract

Highly active antiretroviral therapy (HAART) has considerably improved the prognosis of HIV-infected patients. However, prolonged use of HAART has been related to long-term adverse events that can compromise patient health such as HIV-associated lipodystrophy syndrome (HALS) and nonalcoholic fatty liver disease (NAFLD). There is consistent evidence for a central role of mitochondrial dysfunction in these pathologies. Nucleotide reverse transcriptase inhibitors (NRTIs) have been described to be mainly responsible for mitochondrial dysfunction in adipose tissue and liver although nonnucleoside transcriptase inhibitors (NNRTIs) or protease inhibitors (PIs) have also showed mitochondrial toxicity, which is a major concern for the selection and the long-term adherence to a particular therapy. Several mechanisms explain these deleterious effects of HAART on mitochondria, and evidence points to other mechanisms beyond the “Pol-**γ** hypothesis.” HIV infection has also direct effects on mitochondria. In addition to the negative effects described for HIV itself and/or HAART on mitochondria, HIV-infected patients are more prone to develop a premature aging and, therefore, to present an increased oxidative state that could lead to the development of these metabolic disturbances observed in HIV-infected patients.

## 1. HIV Infection and Antiretroviral Therapy

Human immunodeficiency virus (HIV) infection is a serious public health disorder that affects up to 34 million people in the world [1]. Since this disease was firstly identified and described in the 80s, it has infected at least 60 million people and caused more than 25 million deaths [2]. The introduction of highly active antiretroviral therapy (HAART) has considerably improved the prognosis of HIV-infected patients leading to a significant reduction of HIV-related morbidity and mortality [3]. For these reasons, HIV infection is nowadays considered “just” a chronic infection. There are more than 20 approved antiretroviral drugs classified into five groups according to the mechanisms by which they interrupt the HIV life cycle (Table 1). Current HAART guidelines recommend as first line of treatment two nucleoside/nucleotide reverse transcriptase inhibitors (NRTIs) combined with either one protease inhibitor (PI), one nonnucleoside reverse transcriptase inhibitor (NNRTI) or an integrase inhibitor [4, 5]. Cohort studies and clinical trials have demonstrated that an early initiation of antiretroviral therapy is needed to optimize individual and public health outcomes [6, 7]. However, HAART does not completely eliminate HIV, and treatment must continue throughout patient's life. Prolonged use of HAART has been related to long-term adverse events that can compromise patient health. These deleterious effects have been reported for the majority of antiretroviral drugs and are the most common causes for therapy discontinuation. Some of these disturbances are cardiovascular, neurocognitive, bone, or renal diseases [8–10]. One of the most frequent secondary adverse events caused by HAART is known as HIV-associated lipodystrophy syndrome (HALS). Recent studies also suggest that since the widespread use of HAART, liver diseases represent an important cause of morbidity and mortality in HIV-infected patients. Several studies have demonstrated mitochondrial impairment in HIV-infected patients and especially in those suffering from HALS or fatty liver, suggesting a pivotal role of mitochondria dysfunction in the pathophysiology of these alterations. Thus, this review summarizes the main findings related to the role of mitochondria in HIV, and these two alterations associated HALS and fatty liver. Furthermore, evidence has accumulated suggesting that HIV-infected patients are under chronic oxidative stress and mitochondria dysfunction could contribute to this increased oxidative state. Therefore, we also describe the role of oxidative stress in HIV infection and how different compounds with antioxidant capacities have been studied in an attempt to decrease this oxidative state in a way to ameliorate the deleterious effects of HIV-infection and its metabolic associated disorders.

## 2. HIV and Mitochondria

Mitochondria are intracellular organelles present in all mammalian cells (except red blood cells). They contain an outer membrane, an intramembrane space, an inner membrane, and the matrix where the mitochondrial genome is found. Each mammalian cell contains several hundreds to more than a thousand mitochondria. The size, shape, and abundance of mitochondria vary dramatically in different cell types and may change under different energy demands and different physiological or environmental conditions. The main function of mitochondria is to synthesize ATP through electron transport and oxidative phosphorylation (OXPHOS) in conjunction with the oxidation of metabolites by tricarboxylic acid cycle and catabolism of fatty acids by beta-oxidation. In this sense, the mitochondrion had been, for years, considered to be simply the fuel provider for the most basic energy demands of the cells. However, mitochondria are now recognised as being critical components in the control of multiple key cellular processes, being the main arbitrator in the initiation and execution of apoptosis and, therefore, playing a pivotal role in the determination of life and death of the mammalian cells. In addition, mitochondria are the main intracellular source and immediate target of reactive oxygen species (ROS) [14]. During energy transduction in the mitochondrial electron transport chain, a small number of electrons “leak” to oxygen prematurely, forming the oxygen-free radical superoxide [15]. Under normal conditions, the key site of superoxide formation in the mitochondrial membrane is complex I and the ubiquinone-complex III interface. Given that ROS are highly reactive and short-lived species, it is tempting to suggest that their effects should be greatest in immediate area surrounding their locus of production. Thus, mitochondrial membrane constituents, including the complexes of the respiratory chain and phospholipids constituents, are not only important sources of ROS, but also major targets of ROS attack [16].

Several factors can affect the functionality of mitochondria, such as aging, infections, or certain antiretroviral drugs. These factors can damage the mitochondria, affecting the normal functioning of the cell. Mitochondrial toxicity is a general term that refers to these changes, it can cause different symptoms in the heart, nerves, muscles, pancreas, kidneys, and liver, and it is involved in several disorders. 

It is plausible that chronic infection and inflammation and/or drugs with adverse effects on mitochondrial function would contribute to long-term complications in HIV-infected persons. A large list of clinical manifestations of mitochondrial toxicity has been described within HAART-associated adverse events, which is a major concern for the selection and the long-term adherence to a particular therapy. For example, the increase in tumor necrosis factor (TNF*α*) production, which can directly affect the mitochondrial function, has been observed in patients with HIV infection and under antiretroviral treatment [18]. 

NRTIs have long been considered the main source of HAART-related mitochondrial toxicity. These toxicities can be difficult to reverse and can be life-threatening. The exact pathogenesis underlying NRTI-induced mitochondrial toxicity remains unclear and likely differs for specific NRTI drugs [19]. NRTIs ability to inhibit Pol-*γ*, the DNA polymerase responsible for the synthesis of mitochondrial DNA, has been described. Nevertheless, accumulating evidence points to a more complex relationship between these organelles and NRTIs and other mechanisms beyond the “Pol-*γ* hypothesis” have been raised in the last years suggesting that there are other mechanisms of mitochondrial interference both related and unrelated to mtDNA (reviewed by Apostolova et al., 2011) [17]. Thus, inhibition of mitochondrial RNA expression has been observed in several cell lines exposed to NRTIs [20] which may occur through mtRNA polymerase inhibition or by limitation of the cofactors essential for mtDNA transcription. Some NRTIs also exert a direct inhibitory effect on specific mitochondrial targets unrelated to mtDNA. Thus, AZT inhibits the mitochondrial adenylate kinase and adenosine nucleotide translocator in isolated mitochondria [19]. AZT also promotes oxidative stress (OS) and exerts a direct inhibitory effect on the electron transport chain, thereby diminishing OXPHOS [21, 22]. NRTIs also induce a significant reduction in complex IV activity and a specific inhibition of complex I [21, 23, 24]. *In vivo* studies with AZT demonstrated a disrupted cardiac mitochondrial ultrastructure, diminished expression of mitochondrial cytochrome b mRNA, and the presence of OS in mtDNA. Mitochondrial ROS generation has also been suggested to accompany or even constitute a separate mechanism of NRTI-induced mitochondrial toxicity. It is also important to state that tissue and compound-specific patterns of drug import, compartmentalization, and activation may account, at least in part, for variation in mitochondrial toxicity according to specific NRTIs and/or tissue. Furthermore, not all the drugs of this group exert their deleterious effects on mitochondria with the same intensity. In fact, what became apparent from several *in vitro* studies is that the order of ranking of toxicity differs depending on the cell line and the method used for measuring mitochondrial dysfunction (reviewed by Mallon, 2007) [25]. Thus, AZT and D4T are more toxic to adipocytes while ddC is more toxic to other cell types such as hepatocytes and peripheral blood mononuclear cells (PBMCs). Some observations *in vivo* state that ddC component of antiretrovirals (and possibly ddI) rather than d4T or AZT mediates mtDNA depletion in PBMCs in HIV-infected patients [25]. Other anti-HIV drugs apart from NRTIs seem to also interfere with mitochondria such as PIs and NNRTIs (reviewed by Apostolova et al., 2011) [26]. Interestingly, PIs and NNRTIs do not inhibit Pol-*γ* or reduce mtDNA content. *In vitro* studies revealed an increase in mtDNA in murine adipocytes treated with indinavir (IDV) [27] or treated with nevirapine (NVP) [28]. Recent evidence suggests an interaction of these groups of drugs with mitochondrial targets implicated in the regulation of bioenergetics and apoptosis (deeply reviewed by Apostolova et al., 2011) [29]. Although the clinical repercussion of this interaction is undetermined, it may be relevant in the context of diseases present in long-term HAART patients. However, most findings point to drug- rather than class-specific effects [29].

It is also important to take into account that the progressive aging of the HAART-treated population and the subsequent appearance of age-related diseases are relevant. In fact, it has been demonstrated that antiretroviral therapy through the clonal expansion of mtDNA mutations accelerates mitochondrial aging and, therefore, induces mitochondrial dysfunction [30]. 

Not only antiretrovirals but also HIV itself contributes to mitochondrial dysfunction. HIV *per se* seems to undermine mtDNA and provoke other mtDNA-independent mitochondrial alterations [31]. Thus, HIV and HIV polypeptides (in the absence of ART) have been shown to contribute to mitochondrial dysfunction and apoptosis in CD4+ and CD8+ T cells [32–34]. The study from Morse et al. (2012) also corroborated that HIV infection has direct effects on mitochondria and that immune cell activation and inflammation are involved in this process. In fact, mitochondrial genes were downregulated in PBMCs and adipose tissue from HIV-infected-untreated persons relative to HIV-seronegative and treated patients using a huMITOchip microarray [35]. A study has demonstrated increased plasma mtDNA in acute HIV seroconverters and ART-naive subjects compared with HIV-seronegative controls and long-term nonprogressors, and a positive correlation between plasma HIV RNA and plasma mtDNA was also observed [36]. However, it is worth mentioning that both increases and decreases in mtDNA have been reported in pathogenic conditions as there is not a standard tool for defining what constitutes an abnormal mtDNA quantity, and therefore, data from heterogeneous HIV-infected populations were inconsistent [37–41]. In this sense, there are also other methods to quantify the functionality of mitochondria such as measures of OXPHOS genes and enzymes activities, genes involved in Krebs cycle, lipogenesis, measurement of mitochondrial DNA damage by PCR, and so forth [42]. Thus, depending on the measurement used, the results could be contradictory. 

In summary, it seems quite clear that HAART and HIV itself contribute to mitochondrial dysfunction although the underlying mechanisms remain partially understood. In this context, we cannot forget the importance of patient genetics, the presence of mitochondrial pathologies, and/or pathological settings with compromised mitochondrial function as they can all magnify the adverse effects related to mitochondria.

## 3. Role of Mitochondria in HIV-Associated Fatty Liver Disease

Liver diseases constitute a major health problem nowadays. Among distinct diseases, nonalcoholic fatty liver disease (NAFLD) is a multifactorial condition, which affects both young and adult patients and which is associated with several risk factors (reviewed by Pettinelli et al., 2011) [43]. NAFLD is defined as a chronic inflammatory liver disease that occurs in the absence of alcohol abuse (less than 20 g per day) and also without infection with hepatitis B or C viruses. NAFLD includes a wide spectrum of liver diseases, whose natural history has not yet been fully clarified and ranging from the simple accumulation of fat or fatty liver (process usually mild and reversible) to other phases that are not reversible such as steatohepatitis, fibrosis, cirrhosis, and even hepatocellular carcinoma (HCC). 

Nowadays, NAFLD is one of the most prevalent chronic liver diseases in industrialized countries. This is mainly due to its association with obesity, so it is prevalent in these countries [44]. In fact, NAFLD is the hepatic manifestation of the metabolic syndrome. It is estimated that the prevalence of NAFLD in the general population ranges from 25% to 46%, increasing to 70% in diabetics and 90% among obese [45]. At present, the prevalence of NAFLD continues to rise worldwide, and it is estimated that by 2025 more than 25 million Americans may suffer from NAFLD. It is also suggested that 20% of these patients suffering from NAFLD will develop cirrhosis or HCC if not diagnosed or treated in time [46, 47]. 

Prevalence of NAFLD is higher in individuals with HIV infection (30–40%) than in the general population (14–31%) [48, 49]. In fact, in HIV-infected patients, since the widespread use of HAART, liver diseases represent an important cause of morbidity and mortality [50, 51]. Prospective data from the Data collection on Adverse events of Anti-HIV Drugs (D:A:D) showed that liver-related death was the most frequent cause of non-AIDS-related death, and a strong association between immunodeficiency and risk of liver-related death was demonstrated [51]. In a study analysing 13 cohorts of HIV-infected patients, hepatic mortality accounted for 7.1% of deaths of which 56% were associated with hepatitis, indicating a significant proportion of nonhepatitis-related deaths unrelated to the severity of HIV infection (reviewed by Lemoine et al., 2012) [11, 52]. Recent studies also corroborate these results and suggest a high prevalence of hepatic steatosis and fatty liver in HIV patients, especially those co-infected with HCV or under antiretroviral therapy with NRTIs. Although most of these studies have been carried out in coinfected patients, a recent study found that sixty-seven of 216 (31%) HIV patients without HCV coinfection had NAFLD based on ultrasound evaluation [53]. A very recent study also observed steatosis in 30–40% of HIV-infected patients, associated with increased adiposity and metabolic disorders. This study also demonstrated that the development of fibrosis in these patients was related to age, insulin resistance, and the use of stavudine/didanosine [54]. 

**Figure 1:**
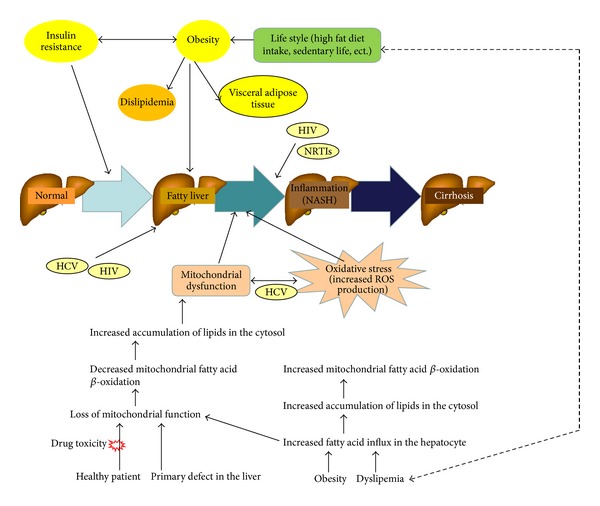
Role of mitochondria in NAFLD initiation and progression (modified from Lemoine et al., 2012 and Grattagliano et al., 2012) [11, 12].

The pathogenesis of NAFLD was previously based on the “two-hit hypothesis” considering steatosis as the result of the first step in the development of NASH [55]. The new “multiple parallel hit model” recently replaced this theory. This new model suggests that insulin resistance and cellular dysfunction play a central role in the development of liver injuries. In fact, insulin resistance is probably the “true first hit” leading to increased hepatic free fatty acid flux from adipose tissue and to hepatocyte oxidative/endoplasmic reticulum stress inducing both steatosis and inflammation [56]. Cellular dysfunction is the consequence of numerous hepatic and extrahepatic mechanisms and mediators. Among them are cytokines derived from the liver but also from gut microflora and adipose tissue (revised by Lemoine et al., 2012) [11] (Figure 1).

In the context of aging and increased prevalence of age-related comorbidities in HIV-infected patients, NAFLD/NASH might represent a facet of this global aging process. In addition, there is consistent evidence for a central role of mitochondrial dysfunction in the pathophysiology of NAFLD. Mitochondria can be directly involved in the first steps of NAFLD either when their function is affected by drugs or by harbouring a primary defect (e.g., resulting from a DNA depletion syndrome). The loss of mitochondrial function will affect fatty acid beta-oxidation, which will lead to the accumulation of nonmetabolized fatty acids in the cytosol. This is further complicated in the presence of obesity and higher circulating levels of fatty acids, which may determine *per se* an increased demand of mitochondrial oxidation. On the other hand, an increase in fatty acid beta-oxidation, as a response to increase hepatocyte influx, can damage normally functional mitochondria through several mechanisms, including uncoupling, induction of the mitochondrial permeability transition (MPT) and OS (Figure 1) (reviewed by Grattagliano et al., 2012) [12]. However, impairment of mitochondrial function also participates in other levels of NAFLD pathogenesis as increasing OS and cytokine production, triggering cell death, inflammation, and fibrosis (reviewed by Grattagliano et al., 2012 and Rolo et al., 2012) [12, 57]. In fact, accumulation of lipids in the hepatocyte impairs the oxidative capacity of the mitochondria, increasing the reduced state of the electron transport chain complexes and stimulating peroxisomal and microsomal pathways of fat oxidation. The consequent increased generation of ROS and reactive aldehydic derivates causes OS and cell death via ATP, NAD, and glutathione depletion, and it finally leads to DNA lipid and protein damage [57]. Through mitochondrial toxicity, NRTIs such as stavudine and didanosine have been identified as factors of NAFLD and fibrosis [58–60]. In this sense, the study of Guaraldi et al. (2008) suggested that NAFLD is common in HIV patients who have traditional risk factors but this study also highlights that treatment with NRTIs is also an important independent risk factor, increasing up to 11% the risk of developing hepatic impairment for each year of use [60]. Some protease inhibitors are also hepatotoxic, in particular full-dose ritonavir and tipranavir [61]. However, the generalized use of only boosting concentrations of ritonavir prevents the liver toxicity. Other protease inhibitors were proposed to induce abdominal lipohypertrophy and to disturb glucose and lipid homeostasis, the underlying conditions of NAFLD/NASH. In addition, some HIV-infected patients, even receiving recently marketed antiretroviral drugs considered as nondeleterious on metabolism and adipose tissue, develop striking abdominal hypertrophy. Efforts have been recently made to assess the clinical relevance of noninvasive tests including the evaluation of mtDNA or mitochondrial functions in peripheral blood mononuclear cells for the diagnosis of antiretroviral-associated toxicity (reviewed by Duong et al., 2005) [62]. In addition, COX subunit I labeling has been suggested to be a valuable tool for the diagnosis of mitochondrial liver disease in HIV patients [63]. Efavirenz (EFV) is the most widely used NNRTIs applied in HAART. Its use has been associated with the development of several adverse events including hepatotoxicity. Recent reports have highlighted features of mitochondrial dysfunction in hepatic cells exposed to clinically relevant concentrations of EFV in human hepatic cells *in vitro* [64]. Clinical concentrations of EFV also induce bioenergetic stress in hepatic cells by acutely inhibiting mitochondrial function. This new mechanism of mitochondrial interference leads to an accumulation of lipids in the cytoplasm that is mediated by activation of AMPK [65]. A recent study has also suggested that autophagy could be involved in the EFV-associated hepatotoxicity, and it may constitute a new mechanism implicated in the genesis of pharmacological liver damage and in the recovery of hepatic homeostasis upon a drug-induced cellular insult [66]. However, and despite the fact that mitochondrial dysfunction seems to be associated with the development of NAFLD in HIV-infected patients, there are other studies carried out in liver biopsies where no association has been found between the presence of NASH and liver mitochondrial function or mitochondrial DNA content [67, 68]. These discrepancies could be due to the fact that it is very difficult to compare *in vitro* versus *in vivo* studies or that these two studies relate NASH and not NAFLD with mitochondrial dysfunction. So far, more studies are needed in this regard to confirm the involvement of mitochondrial dysfunction/damage in the development of NAFLD in HIV-infected patients.

A novel mechanism by which HIV not only enhances HCV replication but also contributes to progression of hepatic fibrosis has been demonstrated [69]. In fact, it has been found that inactivated HIV or its envelope glucoprotein gp120 were capable of upregulating TGB-*β*1 expression. Furthermore, HIV and HCV may independently regulate hepatic fibrosis progression through the generation of ROS, and this regulation occurs in an NF-*κ*B-dependent manner [70]. 

To date, there is no specific treatment against NAFLD both in general and HIV-infected populations. Since insulin resistance is the pathophysiological hallmark of NAFLD/NASH, interventions to improve insulin sensitivity represent one of the main strategies for the therapeutic management of these patients. Thus, one therapeutic option is the use of molecules that sensitize to insulin, and although histological improvements have been observed, the fact is that they do not offer additional long-term benefits. Metformin and glitazones (thiazolidinediones) have been the main insulin-sensitizing molecules tested for the treatment of NASH, but the poor benefit of metformin has been confirmed in a recent meta-analysis [71]. However, metformin treatment was recently found to decrease HCC occurrence and liver-related death in HCV-related compensated cirrhosis [72]. Indeed, the poor safety of glitazones has recently led to their removal from the market. Hepatoprotective agents have been also evaluated, and some of them may be proposed in the case of severe liver disease defined by the presence of NASH and significant fibrosis (F2). The correction of metabolic risk factors, loss of weight, and physical activity seem to be the cornerstones for that [73]. Another treatment option is the use of statins to improve several biological markers of this disease (AST/ALT, lipid profile) although their effects on liver histology are unknown. In most cases, liver transplantation is required. In fact, NAFLD is the third most frequent cause of liver transplant, and some researchers suggest that in the next 10–20 years, NAFLD will be the leading cause of transplants with the high cost that it implies in health services. 

## 4. Role of Mitochondria in HIV-Associated Lipodystrophy Syndrome

HIV-associated lipodystrophy syndrome is characterised by alterations in adipose tissue distribution in association with systemic metabolic complications. The alterations of adipose tissue consist of loss of subcutaneous fat in face, limbs and buttocks [74], accumulation of visceral adipose tissue in abdomen, breast, and dorsocervical regions [75], and lipomatosis, especially in the dorsocervical area (“buffalo hump”). These alterations do not necessarily occur together in the same patient or with the same frequency. Subcutaneous loss of adipose tissue occurs frequently, although more than half patients present a mixed form: loss of subcutaneous together with marked increase in visceral adipose tissue [76]. The metabolic abnormalities comprise hyperlipidaemia, insulin resistance, and lactic acidaemia. HIV-linked lipodystrophy is also associated with atherosclerosis, hypertension [77, 78], and hepatic steatosis [79]. Increased central fat and decreased limb fat are both involved in these metabolic complications as the increased release of cytokines and free fatty acids together with decreased adiponectin production by adipose tissue leads to insulin resistance and triglyceride depots in tissues such as liver, skeletal muscle, and heart. Moreover, aging is also physiologically associated with fat redistribution, OS, and low-grade inflammation, so, it may be a relationship between HALS and aging. Therefore, lipodystrophy together with metabolic alterations contributes to the phenotypes of premature aging observed in these patients, leading to early cardiovascular and hepatic disease risk (deeply reviewed by Caron-Debarle et al., 2010) [80]. 

The prevalence of HALS is about 50%, although reported percentages vary in different studies (20–80%) [76, 81, 82]. Lipodystrophy and especially facial lipoatrophy can erode self-esteem, cause psychological distress, affect quality of life, and lead to depression. It also affects adherence to treatments. However, and up to date, there is no effective treatment for lipodystrophy and only cosmetic surgery seems to be clinically recommended. Because of that, the knowledge of the mechanisms involved in this syndrome will help to develop different strategies to prevent it or, at least, to minimize its metabolic complications. Unfortunately, the molecular basis of this syndrome is not fully understood, although intensive research in recent years has suggested that adipocyte dysfunction is a key point. 

HAART might play an important role in the development of HALS. In this sense, NRTIs favour lipoatrophy whereas viral PIs tend to promote visceral lipohypertrophy and systemic metabolic disturbances. However, recent studies suggest that drugs of the three main classes (NRTIs, IPs, and NNRTIs) act in synergy. Duration of HIV infection, age, and gender may also contribute to the risk of development of HALS. A role for genetics is also probable but has been only partly evaluated. The toxicity of antiretroviral also depends on patients metabolism, which is partly genetically determined [83]. 

The importance of mitochondria in most cells is known. In adipocytes, mitochondria play an important role in their differentiation and function. Preadipocytes mature in two steps: differentiation and then hypertrophy. During the early maturation stage, an increased number of mitochondria are required [84, 85], resulting in small adipocytes, which are highly sensitive to insulin and that secrete high levels of adiponectin [85]. By contrast, older adipocytes increase in size (hypertrophy), lose their functional activities, and become resistant to insulin. They exhibit decreased numbers of mitochondria with impaired functions and secrete less adiponectin [85]. Furthermore, the respiratory chain generates mitochondrial ROS, which could have dual effects on adipocyte differentiation. At physiologically low levels, ROS could act as secondary messengers to activate adipogenesis and lipogenesis, resulting in increased adipocyte number and size, but at higher levels, ROS could inhibit differentiation. High ROS concentrations, which are associated with severe mitochondrial dysfunction, inhibit the expression of the adipogenic factor PPAR*γ* [86] and induce cell apoptosis [84, 87], which could result in lipoatrophy [84].

**Figure 2:**
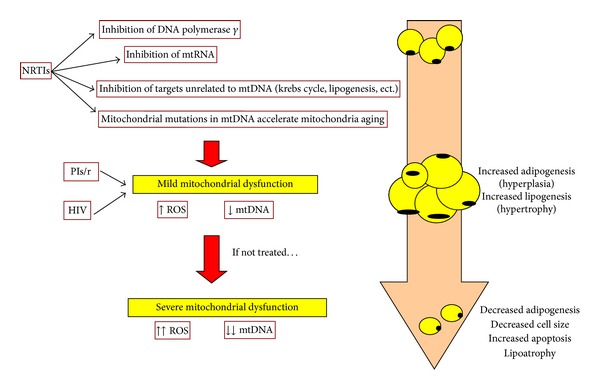
Proposed deleterious impact of mitochondrial dysfunction on adipose tissue (modified from Caron-Debarle et al., 2010) [13].

Longitudinal studies comparing subcutaneous fat in HIV-infected patients before and after HAART initiation have provided important clues. Thus, six to eight months of treatment with NRTIs alter the expression of several key mitochondrial electron transport genes in subcutaneous fat, which leads to increased OS [88]. A reduction in mitochondrial activity and mtDNA has also been observed [89]. In addition, several *in vitro* studies have shown that NRTIs such as zidovudine, stavudine, and didanosine result in mtDNA depletion [90]. Thus, NRTIs exert severe adverse effects on mitochondria, leading to increased ROS production that is probably directly involved in lipoatrophy. This early drug-induced mitochondrial dysfunction could lead to an initial increase in subcutaneous fat before fat loss associated with more advanced and severe mitochondria dysfunction. Mild mitochondrial toxicity leads to an increased production of ROS, which probably activates mitochondrial biogenesis, adipogenesis, and adipocyte hypertrophy. If mitochondrial function is more severely affected, several disturbances in mitochondrial bioenergetics, including increased ROS production, are produced which makes the tissue not able to supply the necessary energy requirements. A decrease in adipocyte size and an increase in adipocyte death is induced, and this could lead to clinical lipoatrophy [13] (Figure 2). Switching patients from stavudine to tenofovir improves fat mitochondrial function and decreases OS [91]. The replacement of these drugs with other NRTIs or NNRTIs or new drugs allows for the partial recovery of lipoatrophy or even causes fat hypertrophy. 

As lipodystrophy has been observed in HIV-infected antiretroviral-naive patients, a possible role for the virus in the development of this syndrome has been suggested. In fact, the HIV infection of macrophages itself could result in low-grade fat inflammation and leads to the release of viral proteins that affect neighboring adipocytes and decreases their differentiation. Even when HIV infection is controlled by HAART, the persistent infection of reservoir fat macrophages, which is probably related to the severity of the initial infection, could help maintain the lipodystrophic phenotype. Furthermore, initial HIV infection associates with increased bacterial translocation through the gut, leading to increased circulating levels of lipopolysaccharide (LPS) [92], which activates macrophages through the TLF-4 receptor and increases cytokine production (reviewed by Caron-Debarle et al., 2010) [80]. In this context, whether mitochondrial function is altered in fat from naive patients remains a matter of debate; either no or mild alterations have been reported in most studies [88, 89, 93] whereas more severe defects were observed by Giralt and colleagues [94]. In this context, a very recent study has demonstrated that HIV infection and antiretroviral therapy could have divergent effects on mtDNA in adipose tissue [35]. Thus, mtDNA content in adipose tissue is decreased in HIV-infected adults receiving HAART, with the most profound reductions seen in patients with lipodystrophy whereas an increase in mtDNA content in adipose tissue was also observed in HIV-infected patients not receiving HAART. Therefore, more work is needed in this regard in order to better elucidate the physiological significance of such duality.

## 5. HIV and Oxidative Stress

As stated before, mitochondria are the main intracellular source and immediate target of ROS. These reactive species, if not counterbalanced by antioxidant defenses, could damage DNA, proteins, carbohydrates, and lipid constituents and compromise cell function leading to the development of a number of diseases such as AIDS [95]. Oxidative stress can result from diminished levels of antioxidants but can also result from increased production of ROS [96]. It is tempting to suggest that mitochondria dysfunction, and more specifically the abnormal mitochondrial ROS generation, could contribute to the increased oxidative state observed in HIV-infected patients. In fact, evidence has accumulated suggesting that HIV-infected patients are under chronic OS. Indications of OS are observed in asymptomatic HIV-infected patients early in the course of the disease. A wide variety of research supports the theory that OS is involved in the progression of HIV disease (reviewed by Pace and Leaf, 1995) [97]. In fact, the role of oxidative stress in HIV disease appears to be quite broad and may involve viral replication but also immune response, apoptosis, disease progression, weight loss, and so forth [97–99]. Tat protein (a protein that plays a pivotal role in both HIV-1 replication cycle and the pathogenesis of HIV-1 infection) induces a progressive elevation of cytoplasmic-free calcium levels, which is followed by mitochondrial calcium uptake and generation of mitochondrial ROS [100]. These mechanisms also contribute to increase HIV transcription. Apart from increased ROS production observed in HIV-infected patients, HIV infection is also able to inhibit glutathione synthesis [101], which is the main endogen antioxidant. HIV-infected persons at all stages of the disease have decreased intracellular glutathione levels. In this sense, perturbations to the antioxidant defence system, including changes in levels of ascorbic acid, tocopherols, carotenoids, selenium, superoxide dismutase, and glutathione, have been observed in various tissues of these patients in comparison with a control population. In addition, plasma zinc and selenium concentrations are very low in HIV-infected patients [102]. In fact, selenium deficiency is common in HIV-positive patients as documented by low plasma and red blood cell levels of selenium, diminished activity of glutathione peroxidase, and low cardiac selenium levels in AIDS hearts. Poor dietary intake and malabsorption could lead to this condition which has important implications for both cardiac and immune functions in HIV-positive patients [103]. Not only HIV monoinfection but also HIV/HCV-coinfection is associated with decreased plasma antioxidants when compared to HIV monoinfection [104]. Furthermore, a decrease of serum total antioxidant status has also been observed in HIV-infected patients on HAART [95]. In this context, it is suggested that HIV infection increases the OS process, and antiretroviral combination therapy increases protein oxidation and preexistent OS [99]. Thus, those patients who had an optimal HAART adherence have significantly higher OS than those with a poor adherence [95]. In general terms, the increase in OS observed after HAART exposure could be due to different biochemical mechanisms but mainly mitochondrial interference caused by NRTIs or by NNRTIs as previously described. It could also be caused by the activation of the P450 hepatic cytochrome by PIs (reviewed by Apostolova et al., 2011) [29]. Therefore, HAART seems to increase OS by increasing the production of ROS, which, in turn, could contribute to the development of several metabolic disturbances associated with HIV-infection such as insulin resistance or cardiovascular events [95, 105–108]. 

Aging is also associated with a chronic low level inflammation [109] and makes the organism more vulnerable to many diseases (i.e., diabetes, obesity, or cardiovascular disease). By 2015, it is expected that more that 50% of the HIV-infected patients in the United States will be ≥50 years [110]. The age of 50 has been considered as a cut-off to discriminate older subjects within HIV-infected patients [111]. The appearance and maintenance of the low-grade inflammation observed in aging is mainly due to the increased OS [109]. Thus, if immune activation and long-term chronic inflammation are major players in the aging process and these processes are more prevalent in HIV-infected patients, even when the infection is well controlled, HIV-infected patients will be more prone to develop a premature aging and, therefore, to present an increased oxidative state that could lead to the development of several metabolic disturbances. 

## 6. Compounds with Antioxidant Capacity as Potential Tools against HIV-Infection and Associated Disorders: Fact or Artifact?

If an increased oxidative stress is observed in HIV-infected patients, actions that could decrease it should be therapeutically beneficial [70, 104]. In fact, attenuation or complete suppression of OS as a way to improve several diseases has flourished as one of the main challenges of research in the last years. Thus, several approaches have been carried out in order to either decrease the high levels of ROS generated or boost the endogenous levels of antioxidants. It is proposed that in the clinical management of HIV-infection with/without hepatitis C (HCV) co-infection, and especially in their early stages, considerable benefit should accrue from antioxidant repletion. In this context, the study of Chandra and colleagues showed that thymoquinone (TQ), an active ingredient of black seed oil from the plant *Nigella sativa* with potent antioxidant properties, is able to inhibit the effect of nelfinavir on augmented ROS production and suppressed SOD levels in pancreatic *β*-cells [112]. A recent study has demonstrated that resveratrol, a SIRT1 activator, attenuates the transactive effects of Tat in HeLa-CD4-long terminal repeat-*β*-gal cells (MAGI) via NAD(+)-dependent SIRT1 activity suggesting that this antioxidant could be a novel therapeutic approach in anti-HIV-1 therapy [113]. Similarly, the study of Touzet and Philips demonstrated a protective effect of resveratrol against protease inhibitor-induced sarco/endoplasmic reticulum stress in human myotubes [114]. Plasma glutathione of HIV-infected patients responded positively and differently to dietary supplementation with cysteine and glutamine. In fact, an increase in total glutathione may be attained by N-acetyl-cysteine (NAC) or glutamine (Gln) supplementation, with NAC acting by increasing cysteine levels and Gln likely acting by replenishing the glycine pool [115]. Other studies demonstrated that gp120 glycoprotein (released during active HIV infection of brain macrophages, thereby generating inflammation and OS which contribute to the development of the AIDS-dementia complex) is toxic to astroglial cells by lipid peroxidation and by altering glutamine release. All the effects of gp120 on astroglial cells were counteracted by NAC, suggesting a novel and potentially useful approach in the treatment of glutamatergic disorders found in these HIV-infected patients [116]. Because plasma zinc and selenium concentrations are very low in HIV-infected patients as previously reported, their replenishment by high dosages seems to be urgent and mandatory particularly in advanced HIV infections. Also recommended is their long-term continuance at high normal levels [102]. However, it is important to note that only selenium-deficient individuals may benefit from selenium supplementation because such supplementation in selenium-replete individuals may even cause higher risk of diseases such as cancer [117]. Furthermore, selenium has a narrow therapeutic window, and there is considerable interindividual variability in terms of metabolic sensitivity and optimal selenium intake. Optimal intake for any individual is likely to depend on polymorphisms in selenoprotein genes that may also affect the risk of disease. Moreover, the baseline levels of each subject could determine the beneficial effect of the selenium intake (reviewed by Pérez-Matute et al., 2012) [118]. Several clinical trials with antioxidant supplements like vitamin E and/or C have also failed to show any significant improvement in markers of OS in other diseases such as type 2 diabetic patients [119], which corroborates that despite the initial positive and beneficial effects observed in many studies not all that glitters is gold. In this context, a recent study showed that antioxidant supplementation may have a protective role in mitochondrial function, with limited effects on the reversal of clinical lipodystrophic abnormalities in HIV-1-infected patients [120]. Thus, whether supplementation with antioxidants will reduce OS in HIV infection is still unknown, and controversial results have been found [99]. 

Other potential strategy to ameliorate OS caused by HAART is switching to a new drug with a better profile. Thus, atazanavir increases bilirubin plasma levels. This increase has been associated with improved endothelial function in patients with type 2 diabetes mellitus [121]. However, controversial results have also been observed in this regard. In virologically suppressed HIV-infected adults on stable HAART, neither total bilirubin nor atazanavir use was associated with improved endothelial function, inflammation, or OS as measured using several biomarkers [122]. So far, there is no available information about the possible relation between OS and other new antiretroviral such as the integrase inhibitors or CCR5 antagonists which limits the chance of using these drugs in an attempt to minimize the impact on OS described for some drugs.

## 7. Concluding Remarks

In the last years, several studies highlighted the central role for mitochondrial impairment in HIV infection and associated disturbances such as NAFLD and HALS. Mitochondrial dysfunction seems to be associated with premature aging in these patients which could contribute to the development of several metabolic pathologies such as cardiovascular events, diabetes, and so forth. The underlying mechanisms that could explain the relationship between mitochondrial dysfunction and HIV, NASH, or HALS are not fully understood despite accumulating evidence points to other mechanisms beyond the Pol-*γ* hypothesis. NRTIs have long been considered the main source of mitochondrial toxicity. In this context, in many parts of the world, persons have been or are currently treated with older NRTIs and remain at risk for overt mitochondrial toxicities. Therefore, mitochondrial toxicity management during HIV therapy has become an important challenge with growing interest in developing new compounds and identifying combinations of available antiretroviral drugs that are clinically effective while eliciting minimal mitochondrial interference in these patients [17]. Therefore, and given that HIV treatment is for life and that antiretroviral drugs have been in use for only 25 years, the fruit of such work could induce invaluable improvement in the quality of life of present and future patients. Other drugs different from NRTIs could also negatively affect the mitochondria. In this regard, evidence points to drug-rather than the class-specific effects on mitochondria. HIV virus itself is also able to induce mitochondrial impairment. Mitochondrial toxicity differs depending on the tissue and the method used for measuring mitochondrial dysfunction. There are also some limitations in this field, since there are not yet adequately validated markers of mitochondrial dysfunction. There is, therefore, an urgent need to investigate in this direction. Finally, it is tempting to suggest that stimulation of mitochondrial function may prevent NAFLD and lipodystrophy development in HIV-infected patients although more studies are also needed in this regard.

## Figures and Tables

**Table 1:** Current employed anti-HIV drug families and reported mitochondrial toxicity (modified from Apostolova et al., 2011) [17].

Drug family (antiretroviral)	Mechanism of action	Mitochondrial dysfunction
Nucleoside/nucleotide reverse transcriptase inhibitor (Abacavir, Didanosine, Emtricitabine, Lamivudine, Stavudine, Tenofovir, and Zidovudine)	Interferes with the HIV reverse transcriptase protein, needed by the virus to make new copies of itself	Inhibition of Pol-*γ*
		Depletion of mtDNA
		Reduction of mtDNA-encoded proteins
		Respiratory chain dysfunction
		Direct inhibition of ETC complexes (I, IV)
		Reduction of ATP levels
		ROS production
		Decrease in *Aψ*_*m*_
		Impairment of ADP/ATP translocase
		Impairment of fatty acid oxidation

Nonnucleoside reverse transcriptase inhibitor (Efavirenz, Etravirine, and Nevirapine)	Stops HIV replication within cells by inhibiting the reverse transcriptase protein	Respiratory chain dysfunction
		Reduction of ATP levels
		ROS production
		Decrease in *Aψ*_*m*_
		Apoptosis

Protease inhibitor (Atazanavir, Darunavir, Fosamprenavir, Lopinavir, Ritonavir, and Saquinavir)	Inhibits the HIV protease activity, a protein required for HIV replication	Inhibition of MPP (mitochondrial protease processing)
		Fragmentation of mitochondrial network
		Increase in mitochondrial Ca^2+^ accumulation
		Apoptosis
		ROS production

Fusion inhibitor (Enfuvirtide)	Prevents HIV from binding to or from entering human immune cells	Unknown

CCR5 inhibitor (Maraviroc)	Prevents HIV from binding to or from entering human immune cells	Unknown

Integrase inhibitor (Raltegravir)	Interferes with the integrase enzyme, which is needed to insert HIV genetic material into human cells	Unknown
